# Real-Time Instance Segmentation of Traffic Videos for Embedded Devices

**DOI:** 10.3390/s21010275

**Published:** 2021-01-03

**Authors:** Ruben Panero Martinez, Ionut Schiopu, Bruno Cornelis, Adrian Munteanu

**Affiliations:** 1 Department of Electronics and Informatics, Vrije Universiteit Brussel, Pleinlaan 2, 1050 Brussels, Belgium; ruben.panero@vub.be (R.P.M.); bruno.cornelis@macq.eu (B.C.); acmuntea@etrovub.be (A.M.); 2 Macq S.A., Rue de l’Aéronef 2, 1140 Brussels, Belgium

**Keywords:** real-time instance segmentation, deep neural network, embedded devices

## Abstract

The paper proposes a novel instance segmentation method for traffic videos devised for deployment on real-time embedded devices. A novel neural network architecture is proposed using a multi-resolution feature extraction backbone and improved network designs for the object detection and instance segmentation branches. A novel post-processing method is introduced to ensure a reduced rate of false detection by evaluating the quality of the output masks. An improved network training procedure is proposed based on a novel label assignment algorithm. An ablation study on speed-vs.-performance trade-off further modifies the two branches and replaces the conventional ResNet-based performance-oriented backbone with a lightweight speed-oriented design. The proposed architectural variations achieve real-time performance when deployed on embedded devices. The experimental results demonstrate that the proposed instance segmentation method for traffic videos outperforms the you only look at coefficients algorithm, the state-of-the-art real-time instance segmentation method. The proposed architecture achieves qualitative results with 31.57 average precision on the COCO dataset, while its speed-oriented variations achieve speeds of up to 66.25 frames per second on the Jetson AGX Xavier module.

## 1. Introduction

Man-made machines sense the real world using multi-modal devices, which offer different measurements of the scene at specific wavelengths or reconstruct the basic geometry of the 3D environment. The intelligent camera systems must perceive and understand the scene by employing efficient computer vision techniques to be able to provide real-time assistance. The most important applications include self-driving vehicles and surveillance systems, which employ a complex mixture of different computer vision techniques, each designed for a specific task, such as detection, segmentation, localization, recognition, registration, segmentation, and tracking, to name a few.

In recent years, the computer vision domain has shifted from statistical methods towards Deep Learning (DL)-based methods [1]. Since image segmentation is a critical component in image analysis and pattern recognition systems, tremendous research efforts were invested in developing novel image segmenting methods by employing innovative Machine Learning (ML) techniques. Semantic segmentation is a first approach for visual scene understanding and focuses on classifying each pixel into a set of object classes [2]. However, instance segmentation is a more challenging task because the goal of instance segmentation is to detect and segment each object instance found in the image [3,4,5,6].

The state-of-the-art approaches on instance segmentation are usually divided into two-stage detectors, e.g., Mask R-CNN [4], and one-stage detectors, such as You Only Look At CoefficienTs (YOLACT) [5], Segment Objects by LOcations (SOLO) [6], and Fully Convolutional Instance-aware Semantic Segmentation (FCIS) [7], to name a few. The two-stage instance segmentation approach consists of first generating a set of candidate Regions-of-Interest (RoIs) and then segmenting and classifying the RoIs. The two stages are generally applied sequentially, and therefore, these methods have difficulties in achieving real-time performance. The one-stage instance segmentation approach focuses on directly generating an explicit localization using a deep neural network. YOLACT [5] proposes to first extract a set of feature maps of different resolutions using a deep convolutional neural network and then further process the feature maps using two parallel branches: (a) the protenet, denoted here as the segmentation head branch, which is used to generate a dictionary of prototype masks, and (b) the prediction head, denoted here as the object detection head branch, which predicts a set of coefficients per instance. SOLO [6] first employs a Fully Convolutional Network (FCN) to distinguish different semantic categories and then performs instance segmentation using two branches: one for category prediction and another for instance mask generation.

In this paper, we propose a novel DL-based real-time instance segmentation method for traffic videos. The proposed method aims to provide real-time performance when deployed in embedded devices and at the same time yield a reliable and close performance compared to the state-of-the-art. The proposed neural network architecture is called SOLACT, which combines the YOLACT [5] and SOLO [6] approaches into a novel design augmented by several key modifications.

The contributions of this paper are as follows: (a) a novel neural network architecture for real-time instance segmentation of traffic video using improved network designs for the object detection and segmentation head branches; (b) a novel post-processing method that ensures a reduced rate of false detection; (c) an improved network training procedure based on label assignment; (d) an elaborated ablation study where lightweight and speed-oriented architectural variations of the basic SOLACT architecture are proposed for deployment on two embedded devices, Nvidia Jetson TX2 [8] and Nvidia Jetson AGX Xavier [9]; (e) an efficient instance segmentation method that outperforms YOLACT [5], the state-of-the-art method for real-time instance segmentation.

The rest of the paper is organized as follows. Section 2 provides an overview of the existing state-of-the-art techniques for instance segmentation. Section 3 describes the proposed method. Section 4 presents the experimental validation. Section 5 draws the final conclusions.

## 2. Related Work

The two-stage instance segmentation approach has gained much popularity as the methods are usually built based on powerful object detection methods such as Faster R-CNN [10] and R-FCN [11]. Mask R-CNN [4] is one of the first object instance segmentation algorithm. It extends the Faster R-CNN [10] architecture for object detection by adding a new network branch for predicting the object masks in parallel with the existing classification and bounding box regression branches. The first stage, called the Region Proposal Network (RPN), is in charge of extracting several features maps of different resolutions. It contains a backbone network typically represented by a Residual Network (ResNet) architecture [12] equipped with a Feature Pyramid Network (FPN) [13]. The second stage consists of a further refinement of the previously detected RoIs, using feature extraction for each candidate box based on RoI pooling, followed by classification and bounding-box regression. Moreover, a parallel branch was added to compute a binary mask for each RoI. The algorithm was further improved in [14], where the Mask Scoring R-CNN method introduces a network block for learning the quality of the predicted instance masks. The block takes the instance feature and the corresponding predicted mask together to regress the mask Intersection over Union (IoU). Similarly, MaskLab [15] was built based on Faster R-CNN [10] and computes three outputs: box detection, semantic segmentation, and direction prediction. Other solutions such as, Constrained Parametric Min-Cut problems (CPMC) [16] and DeepMask [17], further improve the mask prediction. FCIS [7] proposes to detect and segment the object instances jointly and simultaneously based on position-sensitive maps with inside/outside scores. Recently, Mask-Refined R-CNN [18] was proposed by first introducing a feature pyramid for segmentation by establishing a refinement framework on a mask head and by adjusting the stride of the ROI align accordingly, then determining the optimal design scheme by adjusting the size of the input image, the number of feature fusions operations, and the means of feature fusion.

The goal of the one-stage approach is to propose real-time instance segmentation methods by directly generating an explicit localization. YOLACT [5] is one of the fastest state-of-the-art instance segmentation methods based on RetinaNet [19], a one-stage object detector algorithm. RetinaNet employs a ResNet-based backbone architecture, which combines the ResNet [12] and FPN [13] designs to predict a bounding box and a score confidence for each pixel. YOLACT added two more branches to the RetinaNet architecture in order to obtain the segmentation mask for each detection. It computes in parallel a vector of *k* values, using the object detection branch, and *k* common segmentation masks, called prototypes, using the segmentation branch. In the post-processing stage, the detections are thresholded and the high confidence detections are selected as true predictions. The corresponding masks are obtained by multiplying the *k* mask prototypes with the *k*-dimensional mask coefficients vector of the corresponding detection. However, YOLACT’s main disadvantage is that it relies on anchor-based detections, and the segmentation cannot be performed without the bounding boxes branch.

SOLO [6] proposes to predict the segmentation mask and the corresponding score confidence for each location on the image (without bounding boxes) at the same time. Similarly to YOLACT, a ResNet-based backbone architecture that combines the ResNet [12] and FPN [13] designs is employed to compute feature maps of different resolutions for the final part of the network. Each feature map is then downsampled to a lower resolution, where each location (or pixel) is responsible for detecting the object that has the center in it. A two-parallel branches approach is employed to detect the semantic category (with an associated score confidence) and to produce the segmentation. However, the increased complexity heavily affects the method’s speed performance.

Recent works demonstrated that instance segmentation remains a challenging computer vision problem. The work from [20] was motivated by plant image analysis in the context of plant phenotyping and proposed an exemplar-based recursive instance segmentation framework. In [21], the authors proposed a two-stage transfer learning framework for weakly supervised instance segmentation, where the algorithms explicitly discriminate between invalidly and validly generated masks and, in training, only make use of the valid masks to avoid the interference of invalid ones. In [22], the authors studied the problem of aggregating the image-level information of all training images into a large knowledge graph and exploiting semantic relationships from this graph.

## 3. Proposed Method

In this paper, we propose a new instance segmentation method, SOLACT, that provides an improved performance compared with the current state-of-the-art algorithm for image segmentation. SOLACT is then modified for deployment on embedded devices by further increasing the network speed while maintaining a close performance to the basic architecture.

**Figure 1:**
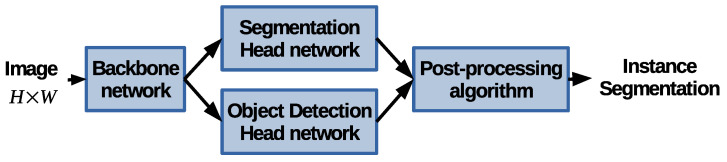
The instance segmentation scheme. The proposed method is obtained by modifying each block.

SOLACT follows the conventional approach, depicted in Figure 1, by first employing a backbone architecture to extract feature maps at different resolutions, followed by two head branches specialized in detecting the class category and the shape of the detected objects, respectively, where the final results are obtained by employing a post-processing algorithm. The proposed method is obtained by modifying each block in Figure 1.

Section 3.1 presents the backbone network design employed in the basic SOLACT architecture. Section 3.2 presents the proposed network design for the segmentation head network. Section 3.3 presents the proposed network design for the object detection head network. Section 3.4 presents the proposed post-processing algorithm for evaluating the network output. Section 3.5 describes the final training details.

### 3.1. Backbone Architecture

The first part of conventional deep neural network algorithms consists of employing a backbone architecture where the weights are pre-trained to perform an image classification task. The most popular backbone architecture is the ResNet architecture [12], where the input patch is processed at five consecutive resolutions, sometimes simply denoted as stages. The corresponding features maps are extracted after processing the patches using bottleneck blocks [12]. The ResNet-18 and ResNet-34 backbone architectures were first proposed using a bottleneck block design with two convolutional layers and, therefore contain a total of 18 and 34 convolutional layers, respectively. The ResNet-50, ResNet-101, and ResNet-152 backbone architectures were later proposed based on a bottleneck block design with three convolution layers with 50, 101, and 152 convolutional layers, respectively. This large variety in available backbone architectures gives the flexibility to choose the optimal architecture corresponding to the target application.

**Figure 2:**
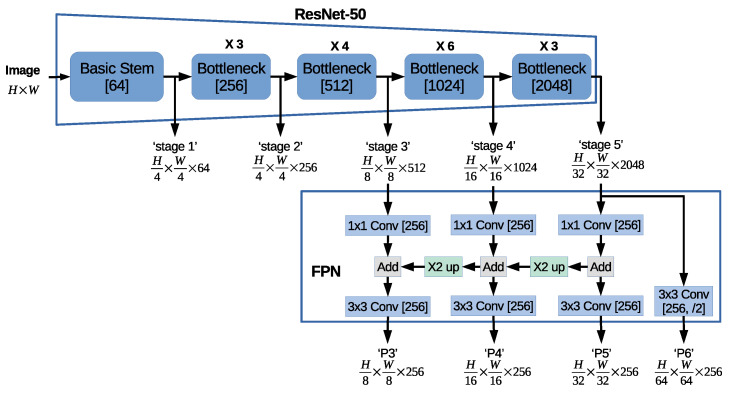
The basic Segment Objects by LOcations (SOLO) combined with You Only Look At CoefficienTs (YOLACT) (SOLACT) architecture employs a ResNet-50-based backbone architecture, which combines the ResNet-50 [12] and FPN [13] architectures.

Recently, ResNet-50 has gained popularity and has become the most used backbone architecture as it provides one of the best performance-complexity trade-offs. The top part of Figure 2 shows the ResNet-50 architectural design, where the input image of size H×W is processed to extract feature maps of different sizes: (1) $\frac{H}{4}\times \frac{H}{4}\times 64,$ denoted as Stage 1; (2) $\frac{H}{4}\times \frac{H}{4}\times 256,$ denoted as Stage 2; (3) $\frac{H}{8}\times \frac{H}{8}\times 512,$ denoted as Stage 3; (4) $\frac{H}{16}\times \frac{H}{16}\times 1024,$ denoted as Stage 4; and (5) $\frac{H}{32}\times \frac{H}{32}\times 2048,$ denoted as Stage 5.

The latest studies show that better results are obtained by further processing the ResNet-based feature maps using the Feature Pyramidal Network (FPN) architecture [13], where the feature maps of different resolutions are processed together in order to efficiently detect the different object sizes. The bottom part of Figure 2 depicts the FPN architecture design [13], which further processes the last three stages from the ResNet-50 network, Stage 3, Stage 4, and Stage 5. A convolution layer with a 1×1 kernel and 256 channels is first employed to unify the number of channels of all feature maps. Note that the object detection head branch uses the same network design to process each feature map. Note that by reducing the number of channels, the network speed is increased. The feature maps with two consecutive resolutions are processed together by first upsampling the lower resolution and then further employing a convolution layer with a 3×3 kernel and 256 channels. Hence, the Stage 3, Stage 4, and Stage 5 feature maps are processed by FPN to obtain the P3, P4, and P5 feature maps, respectively. Note that the Stage 5 feature maps are also processed separately to extract the next lower resolution feature maps, denoted as P6, by employing a convolution layer with a 3×3 kernel, 256 channels, and stride s=(2,2), denoted simply as /2.

Figure 2 depicts the ResNet-50-based backbone architecture used in the proposed basic SOLACT design. In this paper, the neural network is trained using input patches of size H×W, where each input patch is generated by resizing the input image to the H×W=640×640 resolution. Therefore, the backbone network processes the input patch and provides feature maps of the following four resolutions: (i) $\frac{H}{8}\times \frac{H}{8}=80\times 80$ for P3; (ii) $\frac{H}{16}\times \frac{H}{16}=40\times 40$ for P4; (iii) $\frac{H}{32}\times \frac{H}{32}=20\times 20$ for P5; and (iv) $\frac{H}{64}\times \frac{H}{64}=10\times 10$ for P6.

The feature maps generated by the SOLACT backbone are used to predict the classification and segmentation of each foreground object. In the literature, several approaches were proposed to compute the instance segmentation, e.g., by employing a two-stage method where the objects are first detected and then the corresponding segmentation is computed. In this paper, we propose to use a two parallel branches approach, where: (1) the segmentation head branch is in charge of generating the general segmentation maps; and (2) the object detection head branch is in charge of localizing the objects in the input image and computing a confidence score for each class in each feature map cell. Note that the object detection head also provides a vector for every cell used by the post-processing algorithm to generate the corresponding object mask on the cells where an object is present.

The proposed approach generally achieves reduced runtimes compared with the two-stage approach networks, where the inference time remains constant as it does not depend on the number of objects detected in the input image. This property makes the proposed method a good solution for video processing applications where the execution time plays a key role.

### 3.2. Segmentation Head

The segmentation head branch follows the strategy proposed in the ProtoNet branch by YOLACT [5]. The proposed network is designed to process the highest resolution feature maps extracted by the backbone network, i.e., the P3 feature maps, and to compute a set of *k* prototype masks of size $\frac{H}{4}\times \frac{W}{4}.$

**Figure 3:**
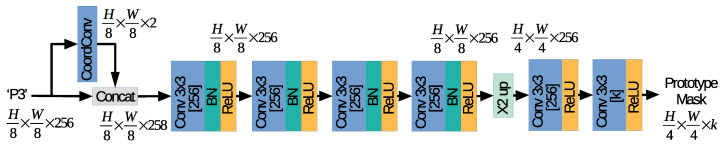
Proposed segmentation head branch.

Figure 3 depicts the proposed architecture used by SOLACT for the Segmentation head branch. In the first part of the network, the P3 feature maps are processed by a Coordinate Convolution (CoordConv) [23], which computes the normalized coordinate positions in the P3 feature map cell. Two feature maps are computed: one contains the horizontal coordinates and the other the vertical coordinates, then concatenated with the P3 feature maps. The second part of the network contains a sequence of four convolutional (Conv) layers with 3×3 kernels and 256 channels, each followed by a Batch Normalization (BN) layer [24] and a Rectified Linear Unit (ReLU) activation layer. The last part of the network contains an upsample layer, which increases the patch resolution from $\frac{H}{8}\times \frac{W}{8}$ to $\frac{H}{4}\times \frac{W}{4}$ and a sequence of two convolution layers with 3×3 kernels, followed by a ReLU activation layer. Note that the first Conv layer after upsampling is equipped with 256 channels, while the second Conv layer after upsampling is equipped with *k* channels so that *k* prototype masks of size $\frac{H}{4}\times \frac{W}{4}$ are computed as the output.

The proposed SOLACT architecture was designed based on the following observations:

(a) To achieve a faster execution time, only the P3 feature maps are used as input for the proposed Segmentation head branch. The feature maps with the highest resolution were selected because they can produce high resolution prototype masks while making use of only one upsample layer. Note that PFN computes the P3 feature maps based on Stage 3, Stage 4, and Stage 5, which results in good quality masks even for small objects.
(b) The utilization of the CoordConv layer represents a novel idea, and it is not part of the original YOLACT [5] design. The proposed design helps to generate prototype masks not only based on the feature maps generated by FPN, but also on pixel locations. Note that each prototype is specialized in a specific segmenting task [5], e.g., for segmenting foreground objects in the left part of the image, and the CoordConv layer is used to reinforce this behavior.
(c) The output of the prototype masks is unbounded, so that the network can output strong activations for the prototypes with high confidence.
(d) The proposed design limits the number of high resolution segmented masks generated by SOLACT by setting k=32. Note that the number of prototypes is independent of the number of objects captured by the image, and it is smaller than the one used in the SOLO architecture [6]. The proposed design helps to reduce the memory footprint of the proposed architecture and the overloading of computational resources.

### 3.3. Object Detection Head

The goal of the object detection head branch is to predict: (i) the location of the objects, (ii) the class of each object, and (iii) a vector of *k* components that represents the mask coefficients used by the post-processing algorithm to generate the final mask, as described in Section 3.4.

**Figure 4:**
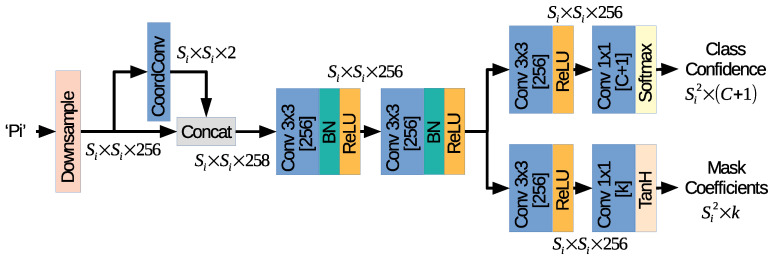
Proposed object detection head branch.

Figure 4 depicts the proposed architecture used by SOLACT for the object detection head branch. The network is used for each feature map Pi, ∀i=3,4,5,6, generated by FPN, i.e., P3, P4, P5, and P6, respectively. However, the trained network weights are shared among all four resolutions. Note that this is a well-known practice [5,6], used in the object detection and image segmentation literature as it allows learning in a homogeneous way for every feature map.

In the first part of the network, each Pi feature map is downsampled to a $S_{i}\times S_{i}$ size and then processed by a CoordConv layer, in a similar way as in the Segmentation Head branch. More exactly, in our case: (i) the P3 80×80 resolution is downsampled to $S_{3}\times S_{3}=60\times 60$; (ii) the P4 40×40 resolution is downsampled to $S_{4}\times S_{4}=30\times 30$; (iii) the P5 20×20 resolution is downsampled to $S_{5}\times S_{5}=14\times 14$; while (iv) the P6 10×10 is maintained, i.e., $S_{6}\times S_{6}=10\times 10$. The second part of the network contains a sequence of two Conv layers with 3×3 kernels and 256 channels, each followed by BN and ReLU layers. The last part of the network consists of two branches. The first branch is specialized in computing the class confidence for C+1 classes using a sequence of two Conv layers: one is equipped with a 3×3 kernel, 256 channels, and a ReLU activation function; and the other one is equipped with a 1×1 kernel, C+1 channels, and a softmax activation function, which computes the confidence as a probability. Similarly, the second branch is specialized in computing the mask coefficients for the *k* prototypes using a sequence of two Conv layers: one equipped with a 3×3 kernel, 256 channels, and a ReLU activation function; and the other one equipped with a 1×1 kernel, *k* channels, and a tanH activation function.

The proposed architecture was designed based on the following design characteristics, which help to improve the network accuracy, as well as increase the speed:

(a) Feature map downsample: Each feature map is downsampled to an $S_{i}\times S_{i}$ grid cell to reduce the execution time with the drawback of limiting the number of possible instances detected, i.e., if there are several very close objects, it will not be possible to distinguish them.
(b) Coordinate convolution: SOLACT follows the approach from SOLO [6] and introduces a pre-processing step based on the CoordConv layer. Thanks to this simple operation, the spatial information is added with a negligible speed impact.
(d) Anchor-free approach: Traditional object detection networks follow the anchor-based approach, where instead of generating a prediction for each cell in the feature maps, several predictions are created, each of them assigned an object size and shape. Such an approach could help to detect different object sizes and shapes; however, recent studies tend to avoid the design complexity of anchor-based detection [25,26,27,28,29]. The anchor-free strategy leverages the training performance, because each location on the feature map is responsible for the detection of objects centered on it, independently of their size or shape. This reduces the complexity, and as a result, the network learns the object features in each location better.
(e) Bounding box-free segmentation: Unlike most the image segmentation networks, SOLACT does not depend on the bounding box prediction to construct the final segmentation masks. This may cause some false mask pieces to get out of the hypothetical bounding box; however, it gives the network much more flexibility for layer pruning and network acceleration.

### 3.4. Post-Processing Algorithm

After training an SOLACT model, a post-processing algorithm is employed to perform the network evaluation. The algorithm is in charge of interpreting the network outputs, deciding where an object was detected, and determining its corresponding segmentation mask. Note that the post-processing algorithm makes it possible to control the reliability of the proposed method and manages the number of false positives so that the method can be employed in a real-time application. However, if the network filters out too many a priori possible instances, then the obvious objects in the images are not detected. Therefore, a balance between the number of detected possible instances and complexity must be reached.

The proposed algorithm contains three parts: (1) initial filtering of the locations with a very low confidence score; (2) mask generation (for the selected locations) and computation of the maskness metric; and (3) final filtering based on non-maximum suppression. Note that the selected final masks of size $\frac{H}{4}\times \frac{W}{4}$ are first upsampled to the input image size, H×W, and then binarized using a threshold of 0.5.

#### 3.4.1. Initial Filtering

The algorithm uses as input the class confidence computed by the object detection head branch; see Figure 4. The initial filtering selects only the feature map cells with the highest probability for an object to be located. Therefore, we first retain only those instances with a class confidence value on the non-background class larger than τ=0.3. From the remaining locations, we further retain only those instances with a class confidence value larger than τ, which gives us the detected object class. This ensures that only a few locations with the highest confidence are selected so that the subsequent computation is accelerated and notably less memory resources are used for real-time applications.

#### 3.4.2. Masks’ Generation and Maskness Computation

Next, we compute a first estimation of the object mask, called soft mask, for each possible location currently detected. The algorithm uses as input: the mask coefficients computed by the object detection head branch (see Figure 4) and the prototype mask computed by the segmentation head branch (see Figure 3). The object’s soft masks are generated by a simple linear combination between the mask coefficients and the prototype mask followed by a sigmoid operation to compute the probability of each pixel.

Experiments showed that some soft masks must be further discarded due to low mask quality or the existence of several masks for the same object. To address the first problem, a mask score confidence called maskness is introduced, which is used in the final filtering step. The maskness is calculated using the following equation:

(1) $$maskness=\frac{\sum_{i}x}{\sum_{i}y},\;\mathrm{where}\left\{\begin{array}{cccc} x=1 & \mathrm{if}\;p_{i}>T_{1}\;\mathrm{and}\; & x=0 & \mathrm{otherwise};\\ y=1 & \mathrm{if}\;p_{i}>T_{2}\;\mathrm{and}\; & y=0 & \mathrm{otherwise}, \end{array}\right.$$

where *i* represents the pixel index in the soft masks, $T_{1}$, $T_{2}$ are two confidence thresholds with $T_{1}>0.5>T_{2}$ ($T_{1}=0.7$ and $T_{2}=0.2$ in our experiments), and $p_{i}\in \left[0,1\right]$ is the probability of the corresponding pixel.

The idea behind the maskness score comes from our observations that a good quality mask contains values from a wide range such that it is able to distinguish between different objects. However, if the mask contains values from a small range, i.e., between the thresholds $T_{2}$ and $T_{1}$, its assigned confidence is reduced.

#### 3.4.3. Final Filtering and Non-Maximum Suppression

The final mask confidence is computed using Equation (2):

(2) $$mask\_confidence=location\_score\cdot maskness^{1.3},$$

where the location score is extracted from the class confidence computed by the object detection head branch (see Figure 4), and the maskness score is computed by Equation (1). The proposed post-processing algorithm was inspired by SOLO [6], where the maskness is computed as the average value of the pixels considered as the foreground, i.e., with a confidence value greater than 0.5. The final confidence score is computed by multiplying the classification score with the maskness [6]. In contrast to SOLO, in this paper, we propose a different strategy where first the maskness is calculated as the ratio between the number of pixels with a confidence value greater than $T_{1}$ and the number of pixels with a confidence value greater than $T_{2}$, as initially it is hard to define a threshold to divide the pixels into background and foreground. Moreover, the final confidence score is computed giving the maskness a higher importance than in SOLO [6]; see Equation (2).

The final filtering algorithm makes use of the mask confidence to filter the remaining masks. This is performed specifically for each class type, i.e., each class has a different confidence threshold, which is set by trial and error on the validation set in order to reduce the false positive rate to less than 3%. The proposed algorithm is able to optimize each class to not discard true positives and at the same time to remove as many false positives as possible.

Finally, the Non-Maximum Suppression (NMS) algorithm is employed to select from the remaining masks only one mask for each object by taking the highest score confidence of all the possible masks for each object. Note that during training, several locations were trained to detect a single object, and if the IoU between the two masks is larger than 0.5, then the two instances are considered to be the same detection, and the mask with the highest score is selected.

### 3.5. Training Details

In this section, we describe in detail the procedure used for training a SOLACT model. Section 3.5.1 presents the proposed procedure employed for ground truth label assignment. Section 3.5.2 presents the loss function formulation. Section 3.5.3 presents the learning rate adjustment.

#### 3.5.1. Labels’ Assignment

The label assignment algorithm decides which feature map and feature map cell must detect and segment the ground truth objects. The ground truth data contain a binary mask for each foreground object in the image, with the same resolution as the input patch, where ones mark pixels corresponding to the object and zeros mark background pixels.

The matrix for object localization is formed by *S* one-hot encoding vectors, where *S* is the number of localization cells. Therefore, each vector contains C+1 coefficients, where only one coefficient is set to “1” to signal the class of the object in the cell. Note that the first index position is signaling the background, i.e., no object is present at the corresponding location, while the remaining *C* coefficients signal an object class. The label assignment algorithm contains the following steps:

Feature map selection: An object may be detected by a specific FPN feature map resolution selected based on its size. Therefore, the small resolution feature maps P5 and P6 are in charge of detecting large objects, while the high resolution feature maps P3 and P4 are in charge of detecting small objects. The relative size of an object, $p_{o},$ computed as the ratio between its mask size and the image size, is used to select the appropriate feature map as follows: (1) if $p_{o}\in \left[0,\;0.005\right),$ then P3 is selected; (2) if $p_{o}\in \left[0.002,\;0.05\right),$ then P4 is selected; (3) if $p_{o}\in \left[0.02,\;0.3\right),$ then P5 is selected; (4) if $p_{o}\in \left[0.1,\;1\right],$ then P6 is selected. The values were selected heuristically by imposing a similar number of instances for each object’s size and feature map. Note that to ensure a smooth progression, the ranges are overlapping, and one or two FPN feature maps may be selected for each object.

**Figure 5:**
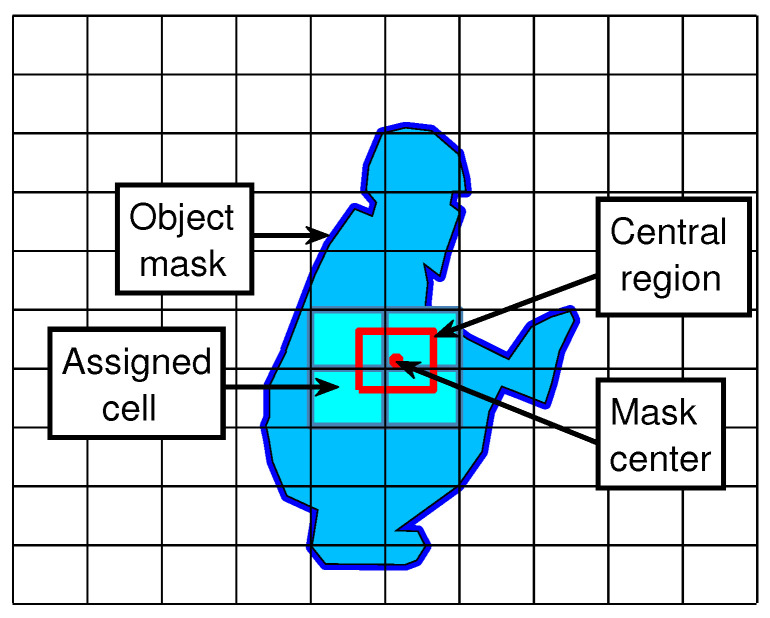
Cells assigned to detect an object. The blue contour marks the object mask. The red dot marks the center of the object mask. The red rectangle marks the center region. The cyan areas mark the cells assigned to detect the object.

Cell assignment: The cells that are assigned to an object are selected based on the center of the object mask as follows: all the grid cells that are intersected by the object’s center region must detect it, as shown in Figure 5. The object’s center region is defined as the rectangle positioned in the center of the object with the following height and width as defined by Equation (3):

(3) $$\begin{array}{cc} center\_region\_width & =0.2\cdot mask_{width}/image_{width}\\ center\_region\_height & =0.2\cdot mask_{height}/image_{height} \end{array}$$

Note that an object can be assigned to several cells because all adjacent locations will have similar features. Therefore, all cells must detect the object so that the post-processing algorithm can select the one with the best mask segmentation; see Section 3.4.

**Figure 6:**
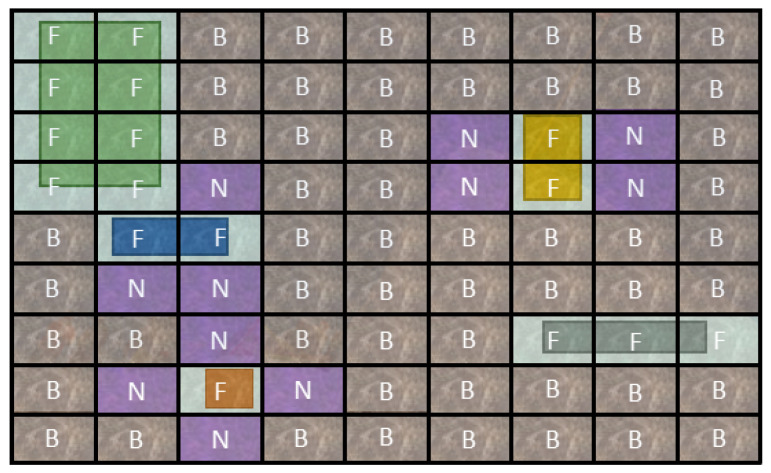
The illustration of the class label assignment for different object sizes. The Foreground cells are marked by “F”. The Background cells are marked by “B”. The Neutral cells are marked by “N”.

Neutral cell assignment: Our experiments show that only one or two feature map cells are assigned to detect small objects, while more than six cells are assigned to detect large objects. In such cases, just a few grids back-propagate the object detection error, and the adjacent cells are assigned as a background region, which can have an impact on the learning performance on the right-side cell. In this paper, when one or two feature map cells are assigned to an object, we propose to mark the adjacent cells as neutral cells. These neural cells do not back-propagate the error, neither as a foreground object nor as background, as depicted in Figure 6. Note that the neutral cells are only taken into account for the calculation of the classification loss as described in the following section.

#### 3.5.2. Loss Function Formulation

The loss function is computed using two terms: one depending on the object localization and classification error and the other depending on the mask segmentation error. The loss is computed as given by Equation (4):

(4) $$L=L_{locate}+\lambda L_{mask},$$

where $L_{locate}$ is the localization loss, $L_{mask}$ is the mask loss, and λ is a weighting term. In this paper, we experimented using λ=6.

$L_{locate}$ is the focal loss introduced in [19] and is defined as follows:

(5) $$L_{locate}\left(p_{t}\right)=-\alpha_{t}{\left(1-p_{t}\right)}^{\gamma }\log\left(p_{t}\right),\;\mathrm{with}\;p_{t}=\left\{\begin{array}{cc} p_{t} & \mathrm{if}\;y_{gt}=1\\ 1-p_{t} & \mathrm{otherwise}, \end{array}\right.$$

where $p_{t}\in \left[0,\;1\right]$ is the predicted classification for each location, $y_{gt}\in \left\{\pm 1\right\}$ is the corresponding ground truth, $\alpha_{t}$ is the class imbalance parameter, and γ is the focusing parameter. Based on our experiments, the parameters in Equation (5) are set to $\alpha_{t}=1$ and γ=1.5, which provide the best performance.

$L_{mask}$ is the loss of predicted masks computed based on the predicted mask and the corresponding ground truth mask. $L_{mask}$ is computed as given by Equation (6):

(6) $$L_{mask}=1-D\left(p,q\right),$$

where D(p,q) is the weighted Dice coefficient introduced in [30] and computed as given by Equation (7):

(7) $$D\left(p,q\right)=\frac{2\sum_{x,y}\frac{p_{x,y}q_{x,y}}{n_{j}}+\epsilon }{\sum_{x,y}{\left(\frac{p_{x,y}}{n_{j}}\right)}^{2}+\sum_{x,y}p_{x,y}^{2}+\epsilon }.$$

where $p_{x,y}$ is the prediction in the soft mask corresponding to the location (x,y), $q_{x,y}$ is the ground truth, $n_{j}$ is the number of different locations for the applicable object *j*, and ϵ is the smooth parameter set to one to avoid the division by zero.

The proposed loss formulation helps the network generate useful prototypes masks and mask coefficients. Note that the Dice loss term allows the network to learn not only the shape of the objects, but also the rest of the object mask, which is predicted as background. This feature has the advantage that we do not depend on bounding boxes to crop the object masks using the post-processing algorithm.

#### 3.5.3. Learning Rate Adjustment

**Figure 7:**
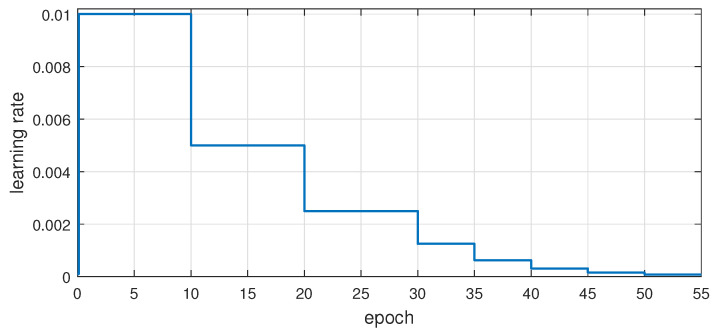
Learning rate adjustment during training.

In this paper, Stochastic Gradient Descent (SGD) is employed as the optimizer with a weight decay of 0.0001, a momentum of 0.9, and using a batch size of 8 images, while training the model for 55 epochs. The learning rate is adjusted based on a static schedule, as depicted in Figure 7. Note that the training starts by using a small learning rate, e.g., $10^{-3},$ to allow the network to adjust the initial weights during only a few first iterations, e.g., the first 0.1 epochs, where the procedure is known as network warm up. Next, the learning rate is set to $10^{-2}$ (initial learning rate) and is further adjusted by halving the value first every 10 epochs up to epoch 30, and then every 5 epochs until epoch 55.

## 4. Experimental Validation

### 4.1. Experimental Setup

COCO dataset:

For instance segmentation, the dataset must provide the objects shapes for every image, which is a very time consuming task when done manually. To build a new dataset for traffic videos, thousands of traffic images and their corresponding annotations are required. One of the most famous datasets for object detection and image segmentation is the COCO dataset [31], which contains 80 types of objects organized into the following 11 super-categories: *person & accessories*, *animal*, *vehicle*, *outdoor objects*, *sports*, *kitchenware*, *food*, *furniture*, *appliance*, *electronics*, and *indoor objects*.

Training set for traffic videos:

The goal of this paper is to propose a novel real-time image segmentation method for traffic monitoring to be deployed on embedded devices. Hence, only the following C=9 traffic classes are extracted from the COCO dataset [31]: *person*, *bicycle*, *car*, *motorcycle*, *bus*, *train*, *truck*, *traffic light*, and *stop sign*. Note that only the images containing an object from any of these classes are used in our experiments. Therefore, the COCO training set, which contains 118,287 images, was pruned to 73,004 images, and the COCO validation set, which contains 5000 images, was pruned to 3086 images. The number of instances of each class on each set is presented in Table 1. Note that the COCO dataset also provides a test set of 20,288 images without publicly available annotations. Therefore, in this paper, the results over the COCO test set are obtained by uploading the instance segmentation results to the COCO server [32] as described below. A resolution of H×W=640×640 was chosen for the input patches as it was considered large enough to be used for the detection of the majority of small and distant objects in the image.

Data augmentation:

To increase the number of training samples and avoid overfitting, two types of data augmentation techniques were applied: (i) horizontal flipping, where the input image is flipped horizontally with a 50% probability; and (ii), random cropping, where the input image is randomly cropped to remove the image borders so that the height and width of the cropped image select between 90% and 100% of the initial height and width, respectively.

Implementation:

The proposed neural network was developed using the open-source object detection and segmentation platform from Facebook AI Research (FAIR) called Detectron2 [33], which was implemented in the Python programming language using the Pytorch [34] open-source deep-learning library. The system ran on a machine equipped with TITAN X [35] Graphical Processing Units (GPUs).

Instance segmentation results:

The accuracy of the proposed network, SOLACT, was compared with the state-of-the-art instance segmentation method YOLACT [5], by means of the COCO metrics results. The following procedure was used to obtain the results:

(1) A network architecture was trained using the training set, and the trained model was saved.
(2) The model was tested on the COCO test set, and the instance segmentation results based on the COCO metrics were saved as a json file.
(3) The json file was uploaded online on the COCO Detection Challenge (Segmentation Mask) website [32], where the numerical results for each class are provided as a scoring output log file.
(4) The results obtained for the C=9 traffic classes were extracted. In this paper, the following six values are reported: (i) the Average Precision (AP) for IoU∈[0.50,0.95] and all areas (small, medium, and large), called AP; (ii) AP for IoU=0.50 and all areas, called AP50; (iii) AP for IoU=0.75 and all areas, called AP75; (iv) AP for IoU∈[0.50,0.95] and small areas, called APs; (iv) AP for IoU∈[0.50,0.95] and medium areas, called APm; (iv) AP for IoU∈[0.50,0.95] and large areas, called AP*ℓ*.

Note that the results for both YOLACT and SOLACT models were obtained using this procedure.

Moreover, the basic SOLACT architecture (presented in Section 3) was further modified, and six other architecture variations were proposed, as described in the ablation study presented in Section 4.4 below. Their performance over the COCO test set was also computed using the procedure presented above.

COCO metric drawbacks:

Recent studies [36] showed that the COCO evaluation metric does not reflect the real accuracy performance as it does not penalize the false positive detection. More precisely, the score detection threshold chosen to generate the final COCO results was set very low, and it provided many false positive detections. In order to implement the model in a real environment application, we proposed to increase the threshold to reduce the number of false detections. Therefore, in this paper, the YOLACT results were generated using the threshold set to 0.4. Furthermore, YOLACT occasionally produces more than one detection for the same object because in the post-processing stage, the NMS operation was applied only to objects of the same category class, e.g., vans were often misclassified as both *cars* and *trucks*. We proposed to correct this behavior by including a function in the YOLACT post-processing procedure, which first computes the IoU between the detected instances, regardless of their class, and then applies NMS on the obtained results.

Model deployment on embedded devices:

The network speed was computed by deploying the trained models on two real-time embedded devices with different hardware specifications: NVIDIA Jetson TX2 having basic hardware specifications [8] and NVIDIA Jetson AGX Xavier having more advanced hardware specifications [9]. The trained models were first exported from the training framework into the open neural network exchange format called ONNX [37]. The converted ONNX model was then optimized to run on the selected embedded device using TensorRT [38], which provides the best computation strategy for the selected device by arranging the code in such a way that parallel calculations are performed as much as possible on the GPU. The network speed was computed based on the average inference time over around 100 images.

### 4.2. Experimental Results

**Figure 8:**
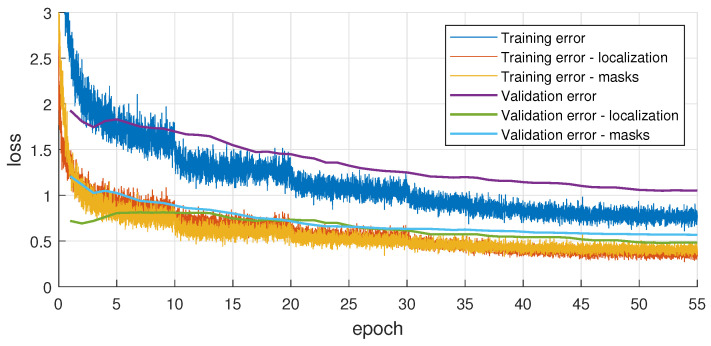
SOLACT model training.

Figure 8 shows the SOLACT training and validation loss. Note that the validation error curve is always very close to the training error curve, which clearly proves that the proposed SOLACT architecture successfully avoids over-fitting.

Table 2 shows the comparison between the instance segmentation results over the COCO test set [31] of nine traffic classes, for the proposed architecture, SOLACT, and YOLACT [5] methods. Note that SOLACT achieves a better average performance, 31.57 AP, compared with YOLACT [5], 30.33 AP. Moreover, for six classes (*person*, *bicycle*, *motorcycle*, *bus*, *train*, and *traffic light*) SOLACT provides a better instance segmentation, while for three classes (*car*, *truck*, and *stop sign*), YOLACT provides a slightly better instance segmentation. Such behavior may be caused by the imbalanced class training; see Table 1.

### 4.3. Qualitative Results

**Figure 9:**
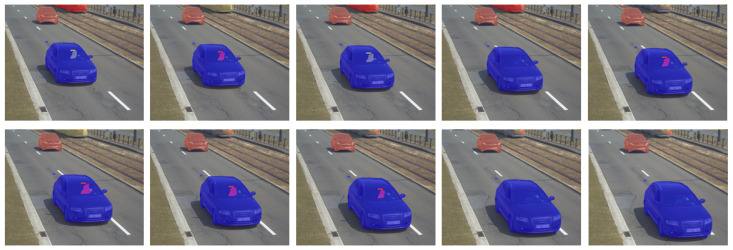
Qualitative instance segmentation results of SOLACT on a real traffic video.

In this paper, the quality of the proposed SOLACT architecture was also tested on a traffic surveillance video. Figure 9 shows the instance segmentation results obtained for a traffic video recorded using a Macq smart traffic surveillance camera [39], kindly provided by Macq S.A./N.V. Note that the sequence was first anonymized in accordance with the General Data Protection Regulation.

Note that the quality of the object masks is quite high and the edges are well adjusted to the object’s shape; although, the resolution of the images captured by the five megapixel camera is about four times larger than the input patch used by the proposed network. Note that for the two cars in the video sequence, the instances detected in the first frame are also detected in all the following frames.

### 4.4. Ablation Study

The final goal of this paper is to achieve real-time performance on embedded devices, while maintaining a competitive performance. Here we propose to further modify the basic architecture design of SOLACT by studying the performance of six different architecture variations of SOLACT, devised based on the following strategies: (1) reduce the complexity of the segmentation head branch; (2) reduce the number of channels in the convolutional layers; (3) design a lightweight version of SOLACT; (4) reduce the input patch size; (5) change the ResNet50-based backbone to a PeleeNet-based backbone [40]; (6) change the ResNet50-based backbone to a MobileNetV2-based backbone [41]. All variations were trained under the same conditions as SOLACT and YOLACT [5]; however, the comparison was performed over the COCO validation set because different COCO accuracy metrics were compared in our attempt to reduce the number of false positive instances detected for each class. Since the annotations on the COCO test set are not publicly available, it is not possible to compute the number of false positive detections on the COCO test set.

**Figure 10:**
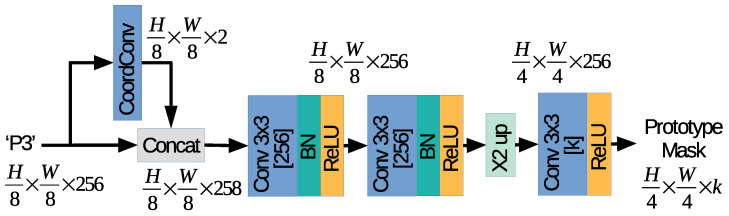
Lightweight segmentation head.

The following six architecture variations of SOLACT are proposed:

(1) Lightweight segmentation head:

In the first network variation, we introduce the following changes with respect to the proposed basic SOLACT network design: the number of convolution layers in the Segmentation head branch is halved. More exactly, the second part of the network contains only two convolutional layers, and the last part contains one convolutional layer, as depicted in Figure 10.

(2) Channel pruning:

The second network variation uses the same number of layers as SOLACT, but the number of channels in the convolutional layer used by FPN to process the feature maps received from the backbone is reduced from 256 channels to 128 channels. The proposed variation aims to heavily reduce the network inference time and study how much the performance is affected by the change.

Our experiments showed that by halving the number of channels only in the Segmentation head branch, a small performance drop is achieved while the inference time is heavily reduced. Therefore, for the remaining architecture variations, this modification is maintained.

(3) Lightweight SOLACT:

The two previously proposed strategies were combined to obtain the third network variation, called lightweight SOLACT. In this case, the segmentation head branch (see Figure 3) contains only one convolution layer with a 3×3 kernel and 128 channels, followed by the BN and ReLU layers. Similarly, the second part of the object detection head branch (see Figure 4) contains only one convolutional layer (followed by the BN and ReLU layers) instead of two convolutional layers.

Our experiments showed that the lightweight SOLACT variation provides a good speed-performance trade-off. Therefore, for the remaining variations, the proposed modifications in the segmentation and the object detection head branches are maintained.

(4) Reduced patch size:

The fourth network variation proposes to train the lightweight SOLACT architecture by reducing the size of the input patches from 640×640 to 512×512.

Therefore, the size of each of the four FPN feature maps is also reduced, so the grid cells on the object detection head branch were also decreased as follows: (i) for P3, from 60×60 to 40×40; (ii) for P4, from 30×30 to 24×24; (iii) for P5, the 14×14 resolution is maintained; and (iv) for P6, from 10×10 to 8×8. Additionally, one more processing block (convolution + BN + ReLU) was introduced in the second part of both the segmentation and object detection head branches.

(5) PeleeNet-based backbone:

The fourth network variation proposes to replace the ResNet-50-based backbone with a PeleeNet-based backbone. More exactly, in Figure 2, the Stage 3, Stage 4, and Stage 5 feature maps are now extracted by a PeleeNet network [40] instead of a ResNet-50 [12] network. Additionally, one more processing block (convolution + BN + ReLU) was introduced in the second part of the object detection head branch.

(6) MobileNetV2-based backbone:

Similarly to the PeleeNet-based backbone variation, the MobileNetV2-based backbone network variation proposes to replace the ResNet-50-based backbone with a MobileNet V2 [41] architecture. The Stage 3, Stage 4, and Stage 5 feature maps are now extracted by a MobileNet V2 network [41], and one more processing block (convolution + BN + ReLU) was introduced in the second part of the object detection head branch.

Table 3 shows the instance segmentation results obtained with the different architecture variations, as well as a comparison between speeds (expressed in frames per second) when deploying these models on the NVIDIA Tegra TX2 [8] and AGX Xavier [9] platforms. The ablation study shows that:

(a) The lightweight SOLACT variation provides the closest performance compared to the basic SOLACT architecture.
(b) The lightweight SOLACT equipped with a PeleeNet-based backbone represents the best choice in terms of speed when deployed on the NVIDIA Tegra TX2 embedded device.
(c) The lightweight SOLACT variation equipped with a MobileNetV2-based backbone represents the best choice in terms of speed when deployed on the NVIDIA AGX Xavier embedded device.
(d) The basic SOLACT architecture remains the best choice in terms of performance.
(e) All proposed architectures achieve real-time performance (more than 30 Frames Per Second (FPS)) when deployed on the NVIDIA AGX Xavier [9].

Table 4 shows the false positive detections of the proposed architectures over the COCO validation set [31]. Note that all proposed architectures achieved a small false positive rate, so we recommend that the models can be used in real-time applications that require high accuracy.

**Figure 11:**
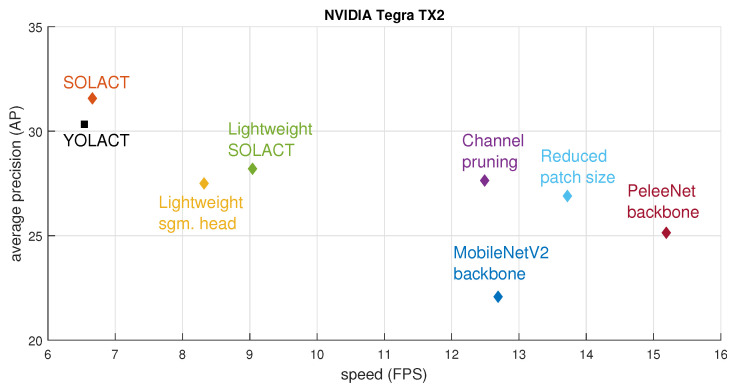
Speed-vs.-performance trade-off, where the models are deployed on the NVIDIA Tegra TX2 [8].

**Figure 12:**
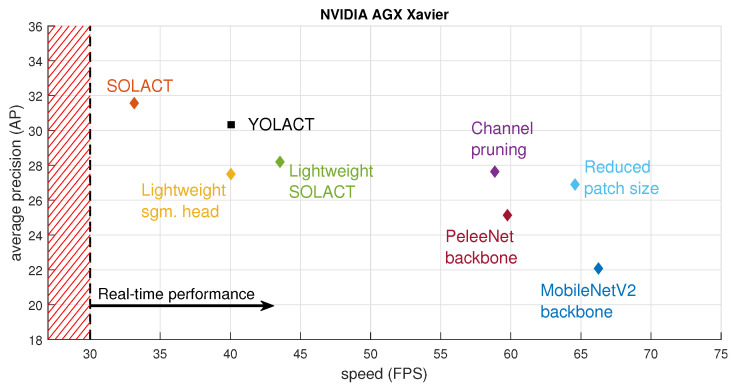
Speed-vs.-performance trade-off, where the models are deployed on the AGX Xavier [9].

Figure 11 and Figure 12 show the speed-vs.-performance trade-off study, where the models were deployed on the embedded devices, Jetson Tegra TX2 [8] and Jetson AGX Xavier [9], respectively. Figure 11 confirms again that for the basic Jetson Tegra TX2 [8] configuration, the PeleeNet-based backbone architecture provides the best speed-vs.-performance trade-off. Figure 12 shows that for the more advanced Jetson AGX Xavier [9] configuration, the reduced patch size architecture provides the best speed-vs.-performance trade-off. Although the MobileNet V2-based backbone architecture achieves the best performance in terms of network speed, its average performance drops too much compared with the basic SOLACT architecture, and therefore, it is not able to provide the a good speed-vs.-performance trade-off.

## 5. Conclusions

The paper proposes a novel real-time instance segmentation method for traffic videos designed for embedded devices. A novel neural network architecture, SOLACT, was proposed by modifying several blocks in the conventional neural network design for instance segmentation. An improved network training procedure was proposed based on a novel label assignment algorithm. SOLACT outperforms the state-of-the-art methods and achieves an average AP of 31.57. The speed-vs.-performance trade-off study was performed for six different SOLACT network variations and demonstrated that: the PeleeNet-based backbone architecture provides the best speed-vs.-performance trade-off when deployed on the Jetson Tegra TX2 [8] module, and the Reduced patch size architecture provides the best speed-vs.-performance trade-off when deployed on the more advanced Jetson AGX Xavier [9] module. All proposed architectures achieved real-time performance when deployed on the NVIDIA AGX Xavier [9] and provided a small false positive rate, which confirmed that the models can be used in real-time applications. Future research will aim at further reducing the complexity and optimizing the performance-complexity trade-offs for the most recent embedded platforms.

## Figures and Tables

**Table 1:** Number of instances per traffic class extracted from the COCO dataset [31].

Class	Number of Instances	
	Training	Validation
*person*	257,253	10,777
*bicycle*	7056	314
*car*	43,533	1918
*motorcycle*	8654	367
*bus*	6061	283
*train*	4570	190
*truck*	9970	414
*traffic light*	12,842	634
*stop sign*	1983	75
TOTAL	352,922	14,972

**Table 2:** Instance segmentation results over the COCO test set [31] using COCO metrics (↑). APs, Average Precision, small areas; APm, AP, medium areas; APl, AP, large areas.

Class	SOLACT						YOLACT [5]					
	AP	AP50	AP75	APs	APm	APl	AP	AP50	AP75	APs	APm	APl
*person*	**32**	51.1	34.7	9.2	37.8	60.5	22.1	37.5	23.5	5.2	22.1	48.6
*bicycle*	**9.9**	21.1	6.8	2.2	11.5	25.6	9.7	25	5.1	1.8	10.9	26
*car*	19.8	29.6	22.3	8.2	35.3	41	**23**	41	22.6	12.5	36.8	44.5
*motorcycle*	**22.8**	40.1	22.2	2.7	16.4	42.7	21.6	43.3	19.5	2.5	14.7	42.2
*bus*	**56.1**	66	62	1.8	36.9	74.2	51.4	62.7	56.8	4.8	29.4	69.7
*train*	**57**	73.5	66.1	12.6	26.2	65.4	55.4	72.4	65.3	11.6	29.3	63
*truck*	15.5	21	17.9	2	12.9	28.6	**22**	33.3	24.8	3.4	17.9	41.5
*traffic light*	**14.2**	26.5	14.3	8.4	31.6	38.6	9.5	18	8.7	4.9	21.8	38.4
*stop sign*	56.8	63.1	62	16.3	58.3	80.7	**58.3**	66.5	65	18.7	60.9	81.6
AVERAGE	**31.57**	43.56	**34.26**	7.04	**29.66**	**50.81**	30.33	**44.41**	32.37	**7.27**	27.09	50.61

**Table 3:** Study of the architecture and scheme variations of SOLACT.

Method	COCO Validation Set						Test Set	Embedded Device	
	Average COCO Metric (↑)							Speed (FPS) (↑)	
	AP	AP50	AP75	APs	APm	APl	AP	Tegra TX2	AGX Xavier
SOLACT	**30.63**	**42.03**	**33.70**	**7.15**	**28.46**	**53.08**	**31.57**	6.66	33.15
Lightweight segmentation head	26.14	35.63	28.29	2.90	23.80	45.87	27.50	8.32	40.04
Channel pruning	25.39	34.53	27.55	3.46	23.93	44.13	27.64	12.49	58.86
Lightweight SOLACT	26.91	37.22	29.23	3.48	24.93	46.54	28.20	9.04	43.54
Reduced patch size	25.18	35.69	26.98	2.77	23.58	45.53	26.90	13.72	64.58
PeleeNet-based backbone	24.20	34.97	26.17	2.16	19.13	46.07	25.14	**15.19**	59.76
MobileNetV2-based backbone	20.82	30.07	22.51	0.79	18.16	40.88	22.08	12.69	**66.25**

**Table 4:** False positive detections (↓) of the proposed architectures over the COCO validation set [31].

Method	*Person*	*Bike*	*Car*	*Motorcycle*	*Bus*	*Train*	*Truck*	*Traffic* *Light*	*Stop* *Sign*
SOLACT	2.78%	1.91%	2.40%	1.91%	2.47%	2.11%	2.42%	2.68%	4.00%
Lightweight segmentation head	2.94%	1.91%	2.66%	2.45%	2.83%	2.63%	1.93%	2.21%	2.67%
Channel pruning	2.68%	1.91%	2.35%	1.63%	2.47%	2.11%	2.90%	2.21%	2.67%
Lightweight SOLACT	2.95%	2.23%	2.45%	2.72%	2.47%	1.58%	2.42%	2.68%	4.00%
Reduced patch size	2.93%	1.91%	2.71%	2.72%	2.12%	1.58%	2.66%	2.37%	2.67%
PeleeNet-based backbone	2.85%	1.91%	3.08%	3.00%	2.83%	1.58%	3.14%	2.05%	4.00%
MobileNetV2-based backbone	2.96%	2.55%	2.97%	3.27%	3.18%	3.16%	3.14%	2.05%	4.00%

## Data Availability

Data sharing not applicable.
